# Emergence and dissemination of multidrug-resistant *Klebsiella pneumoniae* harboring the novel *tmexCD-toprJ* RND efflux pump operon

**DOI:** 10.3389/fcimb.2025.1579880

**Published:** 2025-04-30

**Authors:** Haojin Gao, Bingjie Wang, Meilan Li, Peiyao Zhou, Chunyang Wu, Cailing Wan, Li Shen, Jiana Fu, Weihua Han, Ying Zhou, Fangyou Yu

**Affiliations:** ^1^ Department of Clinical Laboratory Medicine, Shanghai Pulmonary Hospital, Tongji University School of Medicine, Shanghai, China; ^2^ Department of Respiratory Intensive Care Unit, Shanghai Pulmonary Hospital, School of Medicine, Tongji University, Shanghai, China; ^3^ Department of Endocrinology, The First Affiliated Hospital of Wenzhou Medical University, Wenzhou, China

**Keywords:** carbapenem-resistant *Klebsiella pneumoniae* (CRKP), tigecycline (TGC), eravacycline, *tmexCD-toprJ*, hypermucoviscous

## Abstract

The global emergence of multidrug-resistant (MDR) *Klebsiella pneumoniae*, particularly carbapenem-resistant *K. pneumoniae* (CRKP), presents a severe public health threat, limiting available treatment options. Tigecycline and eravacycline, have been considered a last-resort therapeutic against MDR *Enterobacteriaceae*. However, strains were resistant to these antibiotics increased recently. The *tmexCD-toprJ*, a plasmid-encoded resistance-nodulation-division (RND)-type efflux pump, has emerged as a critical factor conferring resistance to tigecycline and eravacycline. In this study, we reported the emergence of 11 CRKP isolates harboring *tmexCD-toprJ*, isolated from two lung transplant patients in a tertiary hospital in eastern China. Most of the isolates (82%) exhibited high-level resistance to tigecycline and eravacycline, along with other common antibiotics. Whole-genome sequencing (WGS) and phylogenetic analysis indicated these strains are not clonal, and resistance phenotypes were associated with the *tmexCD-toprJ* operon and other crucial resistance elements. We also found the *tmexCD-toprJ* operon was located on a conjugative plasmid, sharing high sequence similarity with the operon identified in *Pseudomonas aeruginosa.* Our results showed that the t*mexCD-toprJ*-harboring plasmid is efficiently transferable, which contributes to the dissemination of tigecycline and eravacycline resistance. At the same time, the plasmid can coexist with the *bla_KPC-2_*-carrying plasmid, which may cause multidrug resistance. The emergence of *tmexCD-toprJ*-positive CRKP in lung transplant patients highlights the potential for rapid nosocomial dissemination and reduced treatment efficacy of last-line antimicrobials. Our findings emphasize the need for enhanced genomic surveillance, infection control measures, and alternative therapeutic strategies to combat the spread of *tmexCD-toprJ*-mediated resistance in clinical settings.

## Introduction

Antimicrobial resistance represents a critical global public health challenge, posing significant threats to healthcare systems worldwide. In 2024, the Centers for Disease Control and Prevention (CDC) classified carbapenem-resistant Enterobacteriaceae (CRE) as an urgent public health threat (Antibiotic resistance threats in the United States 2019, 2019). Among these, infections caused by carbapenem-resistant *Klebsiella pneumoniae* (CRKP) are of high concern because of their association with increased mortality and morbidity, and there is an urgent need for effective strategies to address this growing problem (Jin et al., 2021).

Tigecycline, a broad-spectrum antibiotic belonging to the glycylcycline group of antibiotic agents, was designed to overcome the key tetracycline resistance mechanisms, including ribosomal protection and active efflux. It is regarded as a last-line therapeutic option for severe infections caused by multidrug-resistant (MDR) and extensively drug-resistant (XDR) *Enterobacteriaceae*, particularly among Gram-negative pathogens (Stein and Babinchak, 2013). Unfortunately, with increased clinical use, tigecycline-resistant *K. pneumoniae* has been identified in many countries, including China, Japan, Vietnam, and Austria (Sun et al., 2022). Eravacycline, a new synthetic analogue of the tetracycline family recently approved for clinical use, demonstrates strong activity against multidrug-resistant (MDR) Enterobacteriaceae, with notable effectiveness against carbapenem-resistant *Enterobacteriaceae*(CRE) *(* Chakradhar, 2016). However, the eravacycline resistant strains also emerged recently.

The mechanisms by which *Enterobacteriaceae* develop resistance to tigecycline and eravacycline are complex and have not been fully elucidated. Tigecycline resistance in *Klebsiella pneumoniae* is mainly attributed to the overexpression of efflux pump genes such as *acrAB, oqxAB*, and *macAB*, which is caused by mutations in the transcriptional regulators *ramR* and *acrR (* Sheng et al., 2014; Zheng et al., 2018). Additionally, mutations in *rpsJ*, which encodes the tetracycline-targeting ribosomal S10 protein, have been linked to tigecycline resistance (He et al., 2018). Furthermore, mutations in *tet(A)*, another efflux pump gene associated with tetracycline resistance, have also been implicated in reduced tigecycline sensitivity (Yao et al., 2018). Alarmingly, the emergence and rapid spread of plasmid-borne *tet(X3/X4/X5)* confers a high-level tigecycline resistance, posing a significant challenge to its efficacy as a last-line treatment option (He et al., 2019; Sun et al., 2019; Wang et al., 2019).

The horizontal transfer of genetic material via mobile genetic elements, particularly plasmids, plays a crucial role in the emergence and widespread dissemination of multidrug-resistant (MDR) bacteria on a global scale (Carattoli (2013)). Efflux pumps are protein transport systems by which bacteria expel intracellular drugs or toxic substances out of the cell, and are one of the main mechanisms of bacterial resistance to antimicrobial drugs in clinical practice. The *tmexCD-toprJ* operon encodes a novel plasmid-mediated, transferable multidrug-resistant efflux pump of the resistance-nodulation-division (RND) family. The resistance-nodulation-division (RND) superfamily is important to multidrug resistance in Gram-negative bacteria (Li et al., 2015; Du et al., 2018). These RND efflux systems typically operate through the coordinated function of three distinct gene products. Therefore, the horizontal transfer of entire operons encoding RND-type tripartite efflux pumps from chromosomal DNA to plasmids is uncommon (Tauch et al., 2003; Li et al., 2015). This operon, likely originating from *Pseudomonas aeruginosa*, encodes proteins homologous to the RND efflux system, including *tmexC*, *tmexD*, and *toprJ*. The *tmexCD-toprJ* cluster confers resistance or reduced susceptibility to multiple clinically important antimicrobial agents, including tetracyclines such as tigecycline and eravacycline. The emergence of *tmexCD-toprJ* poses a significant threat to clinical management of multidrug-resistant (MDR) infections due to its ability to reduce the efficacy of several antimicrobial agents.

The earliest documented *tmexCD-toprJ*-positive isolate, identified through a GenBank search, was recovered from a chicken in Shandong Province, China, in 2014 (GenBank accession no. QFMD01000298). This paper suggests that the plasmid-encoded cluster emerged at least four years prior to its recognition. Despite its emergence, *tmexCD-toprJ* remains rare among clinical isolates of *Klebsiella pneumoniae* in China, with an estimated prevalence of 0.08% (Lv et al., 2020).

In this study, we report the clinical identification of 11 *Klebsiella pneumoniae* strains harboring *tmexCD-toprJ* isolated from two lung transplant patients. These strains exhibited resistance to both tigecycline and eravacycline, underscoring the clinical relevance of this emerging resistance determinant.

## Methods

### Isolates information

*K. pneumoniae* strains ZY308 and ZY309 were identified in sputum and lavage fluid samples, respectively, from two female patients who underwent lung transplantation in Shanghai. Following the surgery, both patients experienced severe lung infections with recurrent fever. A total of 11 CRKP strains were isolated during their hospitalization.

### Antimicrobial susceptibility testing

The minimal inhibitory concentration (MIC) of *K. pneumoniae* strains ZY308 and ZY309 was determined by broth microdilution method according to the Clinical and Laboratory Standards Institute (CLSI) guidelines. *Escherichia coli* ATCC 25922 was used as the quality control strain. Clinical strains *K. pneumoniae* ZY308 and ZY309 are resistant to eravacycline and tigecycline.

### WGS and bioinformatics analysis

Genomic DNA from the isolates was extracted using a commercial kit (Qiagen, Germany) following the manufacturer’s protocols. Whole-genome sequencing of the 11 CRKP isolates was performed on the Illumina NovaSeq 6000 platform, and two representative strains (*K. pneumoniae* ZY308 and ZY309) were further sequenced using the PacBio Sequel platform. Antimicrobial resistance genes, virulence determinants, plasmid replicon types, serotype predictions, and multilocus sequence typing (MLST) were identified using the Kleborate (Lam et al., 2021), VRprofile2 (Wang et al., 2022), and PlasmidFinder (Carattoli et al., 2014) databases. The related insertion sequences (ISs) and transposons (Tns) were determined with ISfinder (Siguier et al., 2006), while conjugation modules were analyzed by using oriTfinder (Li et al., 2018). Circular plasmid maps were generated with Proksee software (Grant et al., 2023).

The gene environments surrounding *tmexCD-toprJ* in the ZY308 plasmid 1, ZY309 plasmid 1 and pPA033 plasmids were compared using Easyfig software. Sequences were compared using the ClustalW sequence comparison tool in MEGA 11.0. The amino acid sequences of *tmexCD-toprJ* on the three plasmids were aligned using ESPript 3.0 (Robert and Gouet, 2014) and their secondary structures were predicted. To investigate the phylogenetic relationships among the 11 K*. pneumoniae* isolates, genome assemblies were analyzed. The phylogenetic tree of the 11 CRKP isolates was constructed with KSNP4 (Hall and Nisbet, 2023) using ZY309 (GenBank accession GCA_046118685.1) as the reference, and the results were visualized and annotated with the Interactive Tree of Life (iTOL) (Letunic and Bork, 2021).

### String test

To evaluate the mucoviscosity of the *K. pneumoniae* ZY308 and ZY309, the string test was performed as previously described (Yao et al., 2015). Briefly, a positive string test is that a bacterial colony on an agar plate is stretched with an inoculation loop to form a viscous string greater than 5 mm in length.

### Capsule quantification

To evaluate the mucoviscosity of the *K. pneumoniae* ZY308 and ZY309, uronic acid was extracted and quantified as described previously (Wang et al., 2023). An overnight culture grown in Luria-Bertani (LB) media was diluted at 1:100 in media and grown at 37°C for 16 h. Subsequently, 500 µL culture was mixed with 100 µL of 1% Zwittergent 3–12 detergent and heated for 20 min at 50°C, then centrifuged for 5 min at 13,000×g. Next, 300 µl of supernatant was mixed with 1.2 mL absolute ethanol and centrifuged for 5 min at 13,000×g. The pellet was dried and re-suspended in 200 µl of sterile water, to which 1.2 mL of tetraborate solution (12.5 mM sodium tetraborate in sulfuric acid) was added. This was incubated for 5 min at 100°C, followed by immediate cooling on ice for at least 10 min, which was then followed by addition of 20 µl of hydroxyphenyl reagent. After 5 min incubation at room temperature, we determined OD at 520 nm.

### *G. mellonella in vivo* infection model

We applied G. mellonella infection assays to evaluate the pathogenicity of *K. pneumoniae* strains ZY308, NTUH-K2044 (virulence-positive control strain) and HS11286 (virulence-negative control strain). Treatment groups were inoculated with 10 µL of the bacterial suspension containing 1×10^6^ CFU/mL bacteria, while the control groups received 10 µL of normal saline. Each treatment group had at least 30 caterpillars, divided into three Petri dishes, and placed at 37°C. Survival rates were recorded for 3 days with observations every 12 h (Zhou et al., 2024).

### Conjugation assay

According to the analysis with oriTfinder, plasmid 1_ZY308 and plasmid 1_ZY309 were predicted to carry essential conjugative modules. Thus, we used a conjugation assay to test if the resistance plasmid 1_ZY308 and plasmid 1_ZY309 from *K. pneumoniae* ZY308 and ZY309 could be transferred to E. coli EC600 (recipient isolate) as previously described (Zhou et al., 2022). Donors and recipients were incubated to logarithmic phase, mixed in a 1:1 ratio, centrifuged at 8,000g for 1 min, and resuspended in 20 µL of 10 mM MgSO4. The resuspension was plated on LB agar and incubated at 37°C overnight (10 mM). The resuspension was spotted on the LB plate and incubated at 37°C overnight. Subsequently, the serial dilutions were plated in LB plate with appropriate antibiotics (tetracycline, 10 mg/L [tet(A)]; rifampicin, 200 mg/L [E. coli EC600 recipient]). Bacterial identification was performed using matrix-assisted laser desorption/ionization time-of-flight mass spectrometry (MALDI-TOF MS). The number of transconjugants per donor was calculated to determine the conjugation frequency. Polymerase chain reaction (PCR) was performed using PrimeStar (Takara, R045A) to confirm the presence of the tet(A) resistance gene. Primers and PCR amplification condition were described in **Table 1**.

**Table 1:** General features and antimicrobial resistance genes of plasmids in *K. pneumoniae* ZY309.

characteristics	ZY309				
	ZY309_plasmid 1	ZY309_plasmid 2	ZY309_plasmid 3	ZY309_plasmid 4	ZY309_plasmid 5
Accession no.	CP175990.1	CP175991.1	CP175992.1	CP175993.1	CP175994.1
Length (bp)	370,169	131321	112621	15639	5596
GC content (%)	49	53	49	56	51
No. of ORF^a^	400	163	116		
incompatibility group	IncFIB(pNDM-Mar)	IncFII(pHN7A8)/IncFII(pMET)/IncR	IncFIB(pKPHS1)	ColRNAI	/
	IncFII(Yp)				
Conjugal ability					
OriT (start … stop) (bp)	336653.336717	14359.14482	/	/	/
Relaxase (start … stop) (bp)	361221.366461	/	/	/	/
T4CP (start … stop) (bp)	359029.361221	/	/	/	/
T4SS (start … stop) (bp)	69242.96761	12308.25857	/	/	/
	192142.212727	52951.58617	/	/	/
	336105.367200	/	/	/	/
Resistant genes	*ARR-3*	*blaCTX-M-65*			
	*toprJ*	*blaKPC-2*			
	*aac(6’)-Ib-cr*	*blaSHV-12*			
	*aadA16*				
	*aph(3’’)-Ib*				
	*aph(3’)-Ia*				
	*aph(6)-Id*				
	*armA*				
	*blaNDM-1*				
	*dfrA27*				
	*mph(E)*				
	*msr(E)*				
	*sul1*				
	*tet(A)*				
	*tmexC*				
	tmexD				
Virulence factors	/	/	/	/	/

a ORF, Open reading frame.

### Nucleotide sequence accession numbers of *K. pneumoniae* ZY308 and ZY309

The complete nucleotide sequences of the *K. pneumoniae* ZY308 and ZY309 were submitted to GenBank under accession number GCA_046118635.1 and GCA_046118685.1.

### Statistics

Statistical significance was assessed using a two-tailed Student’s t-test and log-rank (Mantel-Cox) test of the GraphPad Prism9 software. P<0.05 was considered statistically significant.

## Results

### *K. pneumoniae* harboring *tmexCD-toprJ* were identified in two Patients with severe lung infection

**Figure 1:**
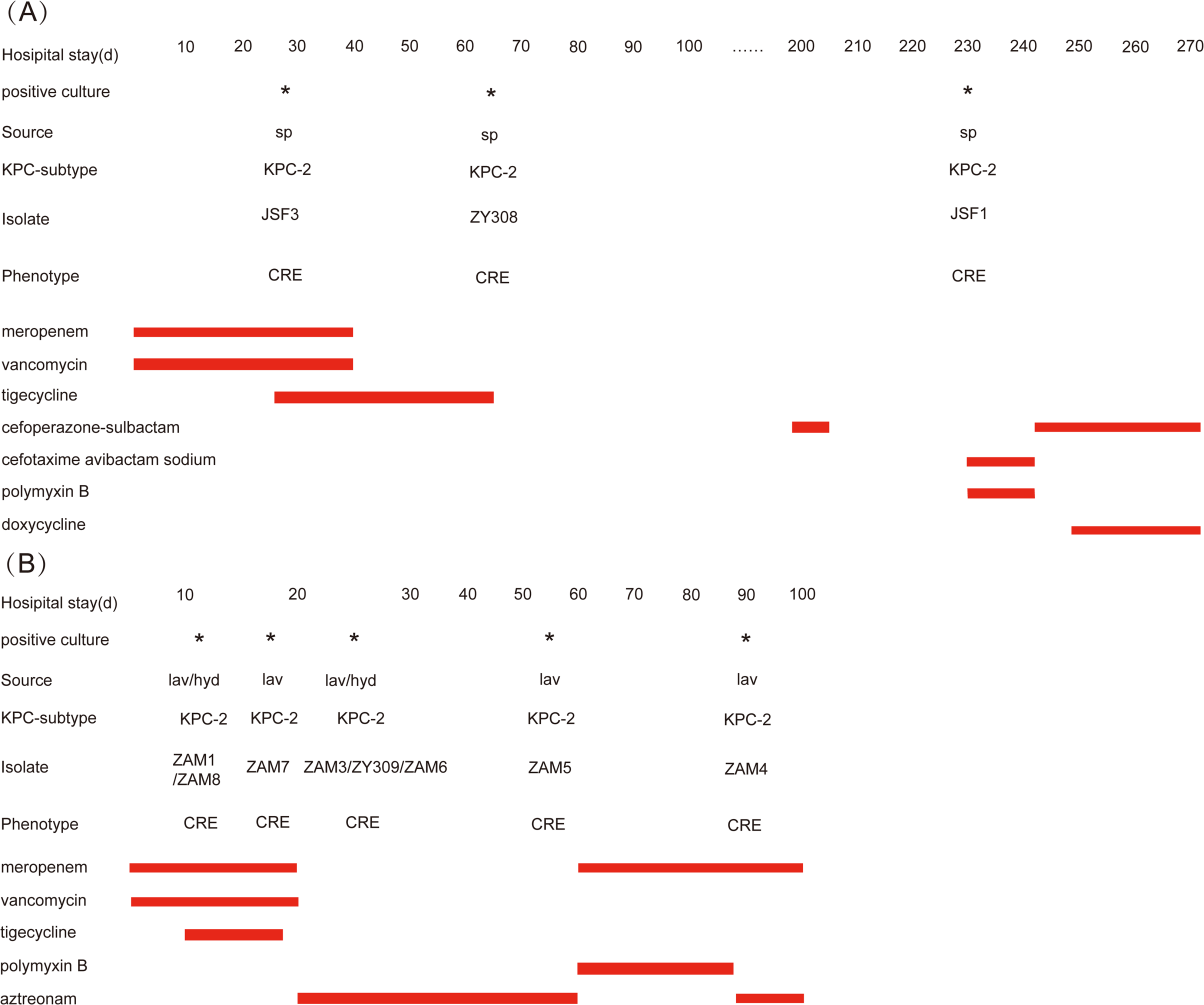
Timeline of infection isolated from patients. **(A)** Time courses of infection and treatment of patient A with multidrug-resistant **(*K*)** *pneumoniae* infection. CRE, carbapenem-resistant *Enterobacterales*; sp, sputum. **(B)** Time courses of infection and treatment of patient B with multidrug-resistant *k. pneumoniae* infection. CRE, carbapenem-resistant *Enterobacterales*; hyd, hydrothorax; lav, alveolar lavage fluid.

During May 2023–January 2024, we identified 11 *Klebsiella pneumoniae* isolates carrying *tmexCD-toprJ* in two female lung transplant patients in the same ward of a tertiary hospital in eastern China. Patient B had eight isolates from bronchoalveolar lavage fluid (BALF) and pleural effusion (PE), while Patient A had three isolates from sputum. The first *tmexCD-toprJ*-positive strain (ZAM8) was detected in May 2023 from PE in Patient B, who underwent continuous anti-infective therapy (**Figure 1A**). On day 11 postoperatively, CRKP ZAM1 (BALF) and ZAM8 (PE) were isolated, with ZAM1 and ZAM8 resistant to all antibiotics except amikacin. We treated the patient with tigecycline and found it ineffective. ZAM1 was found to be resistant to tigecycline and eravacycline by drug susceptibility tests, and was further found to contain *tmexCD-toprJ* operon in both strains by PCR. By day 17, ZAM7, resistant to all antibiotics, was identified, prompting a switch to aztreonam. On day 25, ZAM3, ZY309 (BALF), and ZAM6 (PE) were isolated, all showing multidrug resistance. By day 55, ZAM5 (BALF) was detected, leading to a treatment shift to meropenem (2.5g, q8h, IV) and polymyxin B (0.5g, q12h, IV). On day 90, ZAM4 (BALF) was identified, necessitating another switch to aztreonam. Meanwhile, Patient A, receiving meropenem and vancomycin post-transplant, had CRKP JSF3 (sputum) isolated on day 27, followed by ZY308 (BALF) on day 64, resistant to all antibiotics except amikacin. After initial stabilization and discharge, follow-up therapy included cefoperazone-sulbactam. On day 230, JSF1 (sputum) was detected, requiring polymyxin B and ceftazidime-avibactam treatment, later switched to doxycycline and polymyxin B due to treatment failure. These cases highlight the emergence of *tmexCD-toprJ*-positive CRKP in lung transplant patients and underscore the need for stringent infection control, antimicrobial stewardship, and genomic surveillance to prevent nosocomial dissemination.

### Genomic phylogeny of *tmexCD-toprJ*-harboring *K pneumoniae*

As shown in **Figure 1B**, these 11 *k. pneumoniae* strains belonged to ST11. A total of 11 resistance genes were identified, including the tigecycline-resistant efflux pump gene *tmexCD-toprJ*; the β-lactamase genes *bla*_KPC-1_, *bla*_CTX-M-65_, *bla_NDM-1_*, *bla*_SHV-182_, *bla*_SHV-187_; the aminoglycoside resistance gene *armA*; the tetracycline resistance gene *ramA* and the sulfonamide resistance gene *sul1*. All strains were *tmexCD-toprJ* positive. No virulence factors were identified in any of the 11 strains of *K. pneumoniae*.

**Figure 2:**
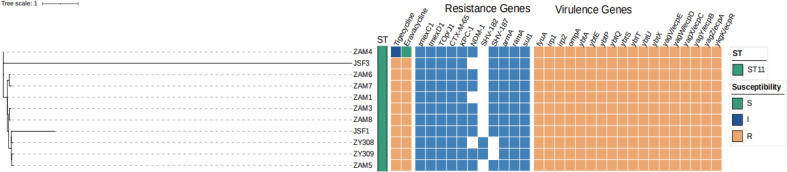
Phylogeny of 11 K*. pneumoniae* isolates based on core genome SNP. Multilocus sequence typing (MLST), capsular (K) type, the distribution of antimicrobial resistance genes, virulence genes and plasmid replicon types are annotated.

From the time courses of infection and treatment of the patient, we could see that these strains appeared after treatment of postoperative anti-infective therapy. The evolutionary tree was constructed using KSNP4, while the evolutionary tree was visualized and annotation information was added via iTOL (https://itol.embl.de/). In the whole-genome single nucleotide polymorphism (SNP)-based phylogenetic tree (**Figure 2**), we can see that both ZAM4 and JSF3 belong to a separate clade each, suggesting that they are distantly related to the other strains. Of the remaining strains, ZAM6 and ZAM7 belonged to clade 1 and both had a SNP difference of 4 compared to the reference strain ZY309. While ZY308, ZY309, JSF1, ZAM3, ZAM5 and ZAM8 were in clade 2, with a maximum SNP difference of 16 and a minimum SNP difference of 1 compared to the reference strain ZY309. Moreover, ZAM1 was similar with ZAM3. We can find that although the resistance genes of these strains are similar, the evolutionary relationship is not from the same clone, and therefore different resistance may be revealed.

### *K pneumoniae* ZY308 and ZY309 exhibited multidrug resistance phenotype

To determine the phenotype and mechanism of these strains, we select two representative strains, ZY308 and ZY309, for the further experiments. MICs for the drug susceptibility test of strains ZY308 and ZY309 were shown in **Table 2**. According to the antibiotic susceptibility test, we found that *K. pneumoniae* ZY308 and ZY309 exhibited similar multidrug-resistant profiles (**Table 2**); they show extensive resistance to all β-lactam antibiotics, including carbapenems, as well as the new β-lactamase inhibitor ceftazidime/avibactam (CZA). Notably, they are resistant to both eravacycline and tigecycline, both of which are considered the last line of defense in CRKP treatment (**Table 2**). In addition, antibiotics that can counteract infections caused by ZY308 and ZY309 are limited; ZY308 is sensitive to amikacin and polymyxin, whereas ZY309 is even sensitive only to polymyxin. However, during the use of polymyxin, patients’ liver function needs to be closely monitored. As this drug can be metabolised and cleared by the liver, prompt adjustment of the treatment regimen is required if liver damage occurs. Therefore, these antibiotic resistance phenotypes are limited by clinical treatment.

**Table 2:** Antimicrobial drug susceptibility profiles.

Antibiotics	MIC(mg/L)										
	ZY308	ZY309	JSF1	JSF3	ZAM1	ZAM3	ZAM4	ZAM5	ZAM6	ZAM7	ZAM8
MEM	≥16(R)	≥16(R)	≥16(R)	≥16(R)	≥16(R)	≥16(R)	≥16(R)	≥16(R)	≥16(R)	≥16(R)	≥16(R)
IPM	≥16(R)	≥16(R)	≥16(R)	≥16(R)	≥16(R)	≥16(R)	≥16(R)	≥16(R)	≥16(R)	≥16(R)	≥16(R)
ETP	≥8(R)	≥8(R)	≥8(R)	≥8(R)	≥8(R)	≥8(R)	≥8(R)	≥8(R)	≥8(R)	≥8(R)	≥8(R)
FOX	≥64(R)	≥64(R)	≥64(R)	≥64(R)	≥64(R)	≥64(R)	≥64(R)	≥64(R)	≥64(R)	≥64(R)	≥64(R)
CAZ	≥64(R)	≥64(R)	≥64(R)	≥64(R)	≥64(R)	≥64(R)	≥64(R)	≥64(R)	≥64(R)	≥64(R)	≥64(R)
CRO	≥64(R)	≥64(R)	≥64(R)	≥64(R)	≥64(R)	≥64(R)	≥64(R)	≥64(R)	≥64(R)	≥64(R)	≥64(R)
CXM	≥64(R)	≥64(R)	≥64(R)	≥64(R)	≥64(R)	≥64(R)	≥64(R)	≥64(R)	≥64(R)	≥64(R)	≥64(R)
FEP	≥32(R)	≥32(R)	≥32(R)	≥32(R)	≥32(R)	≥32(R)	≥32(R)	≥32(R)	≥32(R)	≥32(R)	≥32(R)
CZA	4(R)	4(R)	≥64(R)	≥64(R)	≥64(R)	≥64(R)	≥64(R)	≥64(R)	≥64(R)	≥64(R)	≥64(R)
CSL	≥64(R)	≥64(R)	≥64(R)	≥64(R)	≥64(R)	≥64(R)	≥64(R)	≥64(R)	≥64(R)	≥64(R)	≥64(R)
TZP	≥128(R)	≥128(R)	≥128(R)	≥128(R)	≥128(R)	≥128(R)	≥128(R)	≥128(R)	≥128(R)	≥128(R)	≥128(R)
AMC	≥32(R)	≥32(R)	≥32(R)	≥32(R)	≥32(R)	≥32(R)	≥32(R)	≥32(R)	≥32(R)	≥32(R)	≥32(R)
AMK	8(S)	≥64(R)	8(S)	≥64(R)	≥64(R)	≥64(R)	≥64(R)	≥64(R)	≥64(R)	≥64(R)	≥64(R)
LVX	≥8(R)	≥8(R)	≥8(R)	≥8(R)	≥8(R)	≥8(R)	≥8(R)	≥8(R)	≥8(R)	≥8(R)	≥8(R)
TGC	≥8(R)	≥8(R)	≥8(R)	≥8(R)	≥8(R)	≥8(R)	4(I)	≥8(R)	≥8(R)	≥8(R)	≥8(R)
ERV	4(R)	4(R)	1.5(R)	2(R)	1.5(R)	3(R)	1(S)	1.5(R)	2(R)	1.5(R)	2(R)
COL	0.5(S)	0.5(S)	1(S)	0.5(S)	2(S)	1(S)	≥8(R)	≥8(R)	2(S)	4(R)	0.5(S)
SXT	≥320(R)	≥320(R)	≥320(R)	≥320(R)	≥320(R)	≥320(R)	≥320(R)	≥320(R)	≥320(R)	≥320(R)	≥320(R)

MIC, minimal inhibitory concentration; MEM, meropenem; IPM, imipenem; ETP, ertapenem; FOX, cefoxitin; CAZ, ceftazidime; CRO, ceftriaxone; CXM, cefuroxime; FEP, cefepime; CZA, ceftazidime-avibactam; CSL, cefoperazone/sulbactam; TZP, piperacillin-tazobactam; AMC, amoxicillin/clavulanic acid; AMK, amikacin; LVX, levofloxacin; TGC, tigecycline; ERV, eravacycline; COL, polymyxin E; SXT, trimethoprim/sulfamethoxazole.

### *Klebsiella pneumoniae* ZY308 and ZY309 revealed hypermucoviscous phenotype and their virulence characteristics

**Figure 3:**
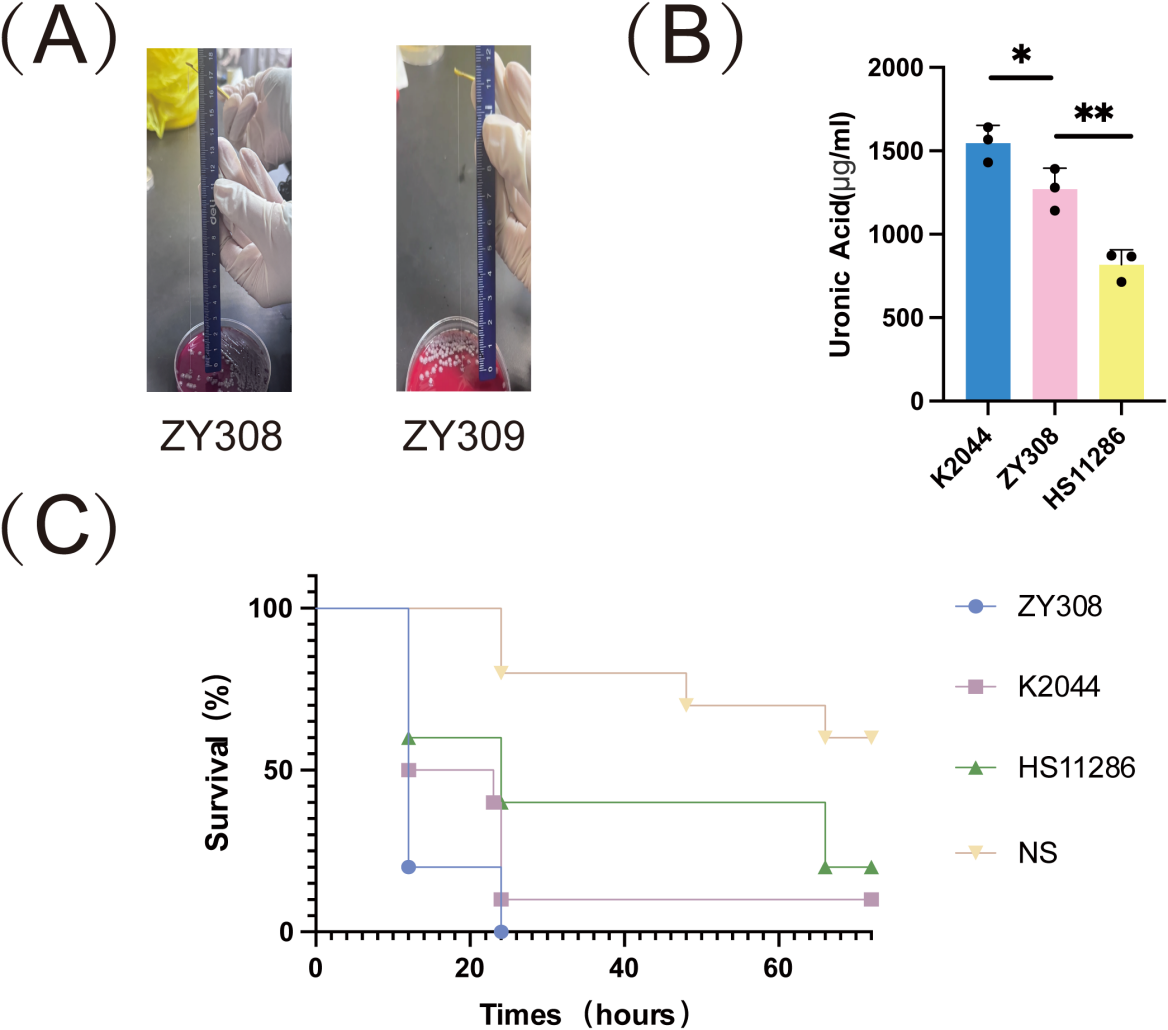
The virulence phenotype of ZY308 and ZY309. **(A)** Results of string tests. Generally, a string 5 mm or longer was defined as positive. **(B)** Capsule production of ZY308 measured by uronic acid levels. **(C)** The survival curves of infected by ZY308, NTUH-K2044 and HS11286. NS (normal saline). Unpaired two-sided Student’s t-test was performed for uronic acid production and survival rate of serum resistance assay. A log-rank (Mantel-Cox) test was performed for the survival curves.

Capsules are polysaccharide matrices encapsulated on the bacterial surface, also known as “K” antigens, which are the basis for *K. pneumoniae* serotyping and are the most essential and widely studied virulence factors for pathogenesis of *K. pneumoniae*. we aimed to explore whether *K. pneumoniae* ZY308 and ZY309 poses hypervirulent features. *K. pneumoniae* strain HS11286 (classical *K. pneumoniae*, cKp, ST11) was used as virulence-negative control strain, and NTUH-K2044 (ST23, KL1) was used as a virulence-positive control strain. *K. pneumoniae* ZY308 appeared as a white mucus form with a positive string test result (**Figure 3A**). As shown in **Figure 3A**, string tests showed that ZY308 and ZY309 produced viscous filaments (173 mm and 113 mm), which were postive results, indicating the presence of the hypermucoviscous phenotype in these two strains. In addition, capsule formation was quantified by measuring uronic acid. As shown in **Figure 3B**, ZY308 and ZY309 produced uronic acid less than NTUH-K2044 and showed an equivalent level to HS11286. Furthermore, we applied *G. mellonella* larvae infecting model to calculate pathogenicity. At 3-day post-infection, ZY308 (0%), and NTUH-K2044 (positive control, 10%) showed comparable virulence resulting in low survival, while HS11286 (negative control, 20%) exhibited relatively higher survival, suggesting a high level of pathogenicity of ZY308 (**Figure 3C**).

### Molecular characteristics of ZY308 and ZY309

**Figure 4:**
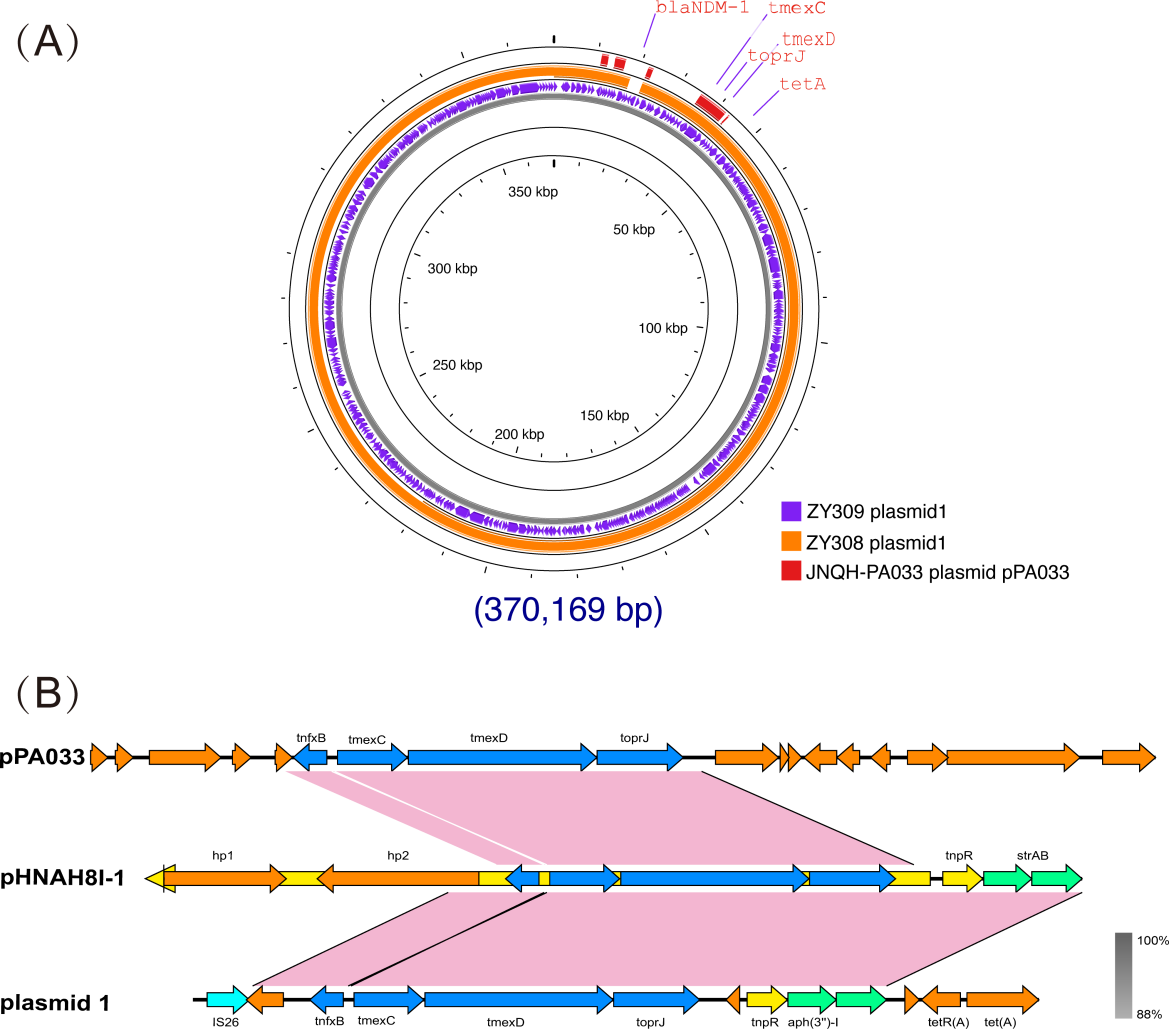
Comparative analysis of plasmid 1_ZY308 and plasmid 1_ZY309 with other reference plasmids using Proksee. A BLAST search was performed to identify the similar plasmids in GenBank. **(A)** Schematic map of plasmid 1_ZY309. The sequence alignment between plasmid 1_ZY309 and plasmid 1_ZY308 (GenBank accession No. CP176043.1, IncFIB(pNDM-Mar)/IncFII(Yp)) is shown in the circle of orange. The sequence alignment between plasmid 1_ZY309 and JNQH-PA0333 plasmid pPA033 (GenBank accession no. CP003223.1) is shown in the circle of red. **(B)** Genetic environment of *tmexCD-toprJ* operon located on plasmid 1 compared with other plasmids.

To explore the mechanism of drug resistance phenotype. We applied WGS to ZY308 and ZY309. It was found that all of them have *bla*_kpc-2_, and even some bacteria contain both *bla*_KPC-2_ and *bla*_NDM-1,_ which may be the reason why they are generally resistant to β-lactams. All 11 *Klebsiella pneumoniae* isolates analyzed in this study harbored the *tmexCD-toprJ* efflux pump operon, with its high expression identified as the primary mechanism mediating resistance to tigecycline and eravacycline. Notably, genomic analysis revealed the absence of classical virulence genes in these isolates, consistent with the virulence assay results for strain ZY308. Whole-genome sequencing (WGS) identified a 5.6 Mb chromosomal genome and six plasmids in ZY308. Among these, ZY308_plasmid 1 was a fully assembled 375 kb plasmid carrying 15 distinct classes of antimicrobial resistance genes (ARGs). In contrast, ZY308_plasmid 3 harbored only *bla*_KPC-2_ and *bla*_SHV-12_, while ZY308_plasmid 4 (46 kb) encoded *bla*_CTX-M-65_ and *bla*_SHV-12_. The remaining plasmids (ZY308_plasmid 2, ZY308_plasmid 5, and ZY308_plasmid 6) lacked ARGs and conjugation-associated elements. Similarly, ZY309 comprised a 5.6 Mb chromosomal genome and five plasmids. ZY309_plasmid 1, a fully assembled 379 kb plasmid, harbored 16 distinct ARGs, including *tmexCD-toprJ* and *bla*_NDM-1_. ZY309_plasmid 2 (135 kb) contained *bla*_CTX-M-65_, *bla*_KPC-2_ and *bla*_SHV-12_, while ZY309_plasmids 3, 4, and 5 lacked ARGs and conjugative elements. Comparative plasmid analysis demonstrated that ZY308_plasmid 1 exhibited 98.76% sequence similarity to ZY309_plasmid 1, with both belonging to the IncFIB(pNDM-Mar)/IncFII(Yp) incompatibility group. The key genetic distinction between these two plasmids was the presence of *bla*_NDM-1_ in ZY309_plasmid 1, which was absent in ZY308_plasmid 1 (**Figure 4A**). These findings highlight the genomic plasticity of plasmid-mediated tigecycline resistance and the potential for horizontal dissemination of tmexCD-toprJ among clinical *K. pneumoniae* isolates.

Previous studies have suggested that the *tmexCD-toprJ* operon may originate from *Pseudomonas aeruginosa (* Lv et al., 2020; Dong et al., 2022). So, we also compared ZY308_plasmid 1 and ZY309_plasmid 1 with the typical *Pseudomonas aeruginosa* drug-resistant plasmid pPA033 (GenBank accession no. CP003223.1), and the result shows that the *tmexCD-toprJ* operon on the three plasmids are matched(**Figure 4A**). Plasmid pHNAH8I-1(GenBank accession no. MK347425.1) is a plasmid carrying *tmexCD-toprJ* operon, also isolated from *Klebsiella pneumoniae*. The *tmexCD-toprJ* cluster was located within transposon Tn5393 as part of an insertion that disrupted the transposase in plasmid pHNAH8I-1 (Chiou and Jones, 1993; Lv et al., 2020; Sun et al., 2020). In ZY308_plasmid 1, the hp2 gene located upstream of this cluster was truncated by insertion sequence IS26 with other parts remaining the same, indicating that the *tmexCD-toprJ* cluster in ZY308_plasmid 1 may have evolved from plasmids such as pHNAH8I-1by genetic recombination(**Figure 4B**). Plasmid pPA033, a plasmid containing the *tmexCD-toprJ* operon in *Pseudomonas aeruginosa*, is compared with the genetic environment of *tmexCD-toprJ* operon located on ZY308_plasmid 1 in **Figure 4B**.

### Mulidrug-resistant phenotype could be transferred from ZY308 and ZY309 to other isolates

Based on the evolutionary tree results we found that ZY308 and ZY309 are not on the same clade, indicating that they are not the same clone. However, both strains harbor the *tmexCD-toprJ* efflux pump operon, which was identified on plasmid 1 in both isolates using ResFinder. In both ZY308 and ZY309, plasmid 1 was classified as an IncFIB-type conjugative plasmid, carrying four complete conjugation-associated modules (**Tables 1**, **3**), confirming its potential for horizontal transfer.

**Table 3:** General features and antimicrobial resistance genes of plasmids in *K. pneumoniae* ZY308.

Characteristics	ZY308					
	ZY308_plasmid 1	ZY308_plasmid 2	ZY308_plasmid 3	ZY308_plasmid 4	ZY308_plasmid 5	ZY308_plasmid 6
Accession no.	CP176043.1	CP176044.1	CP176045.1	CP176046.1	CP176047.1	CP176048.1
Length (bp)	365,584	113927	87028	45291	15639	5596
GC content (%)	49	49	54	52	56	51
No. of ORF^a^	391	119	107	52	16	10
incompatibility group	IncFIB(pNDM-Mar)	IncFIB(pKPHS1)	IncFII(pHN7A8)	IncFII(pMET)	ColRNAI	/
	IncFII(Yp)		IncR			
Conjugal ability						
OriT (start … stop) (bp)	318992.319056	/	46668.46753	6951.7074	/	/
Relaxase (start … stop) (bp)	343569.348809	/	/	/	/	/
T4CP (start … stop) (bp)	341368.343569	/	/	/	/	/
T4SS (start … stop) (bp)	49671.77190	/	19119.22824	4900.18449	/	/
	172571.193156	/	/	/	/	/
	318444.349548	/	/	/	/	/
Resistant genes	*ARR-3*		*blaKPC-2*	blaCTX-M-65		
	*toprJ*		*blaSHV-12*	blaSHV-12		
	*aac(6’)-Ib-cr*					
	*aadA16*					
	*aph(3’’)-Ib*					
	*aph(3’)-Ia*					
	*aph(6)-Id*					
	*armA*					
	*dfrA27*					
	*mph(E)*					
	*msr(E)*					
	*sul1*					
	*tet(A)*					
	*tmexC*					
	*tmexD*					
Virulence factors	/	/	/	/	/	/

a ORF, Open reading frame.

To assess the transferability of *tmexCD-toprJ*, bacterial conjugation and electroporation experiments were performed. The results demonstrated that ZY308_plasmid 1 and ZY309_plasmid 1 were successfully transferred to *Escherichia coli* EC600, with average conjugation frequencies of 5.768×10⁻^5^ and 1.82×10⁻^5^, respectively. Notably, antimicrobial susceptibility testing revealed that the transconjugants exhibited significantly increased resistance, particularly to carbapenems, further confirming that carbapenem resistance determinants were co-transferred alongside the plasmids.

These findings indicate that *tmexCD-toprJ*-mediated tigecycline resistance is highly transmissible via conjugative plasmids, raising concerns about its potential spread in clinical settings. Based on these results, we hypothesize that additional *K. pneumoniae* strains may have acquired this resistance operon through the horizontal transfer of conjugative plasmids, further contributing to the dissemination of tigecycline resistance among CRKP isolates.

## Discussion

The global prevalence of multidrug-resistant (MDR) *Klebsiella pneumoniae* has been on the rise over the past decade, especially carbapenem-resistant *Klebsiella pneumoniae* (CRKP), which poses a major challenge to global public health. Tigecycline has long been considered the last line of defense in the treatment of infections caused by MDR *Klebsiella pneumoniae*. Tigecycline resistance in *Klebsiella pneumoniae* is mainly caused by overexpression of efflux pump genes (e.g., *acrAB, oqxAB, macAB*), which usually result from *ramR* and *acrR* mutations (Sheng et al., 2014; Zheng et al., 2018). In addition, *rpsJ* mutations (affecting the ribosomal S10 protein) (He et al., 2018) and *tet(A) (* Yao et al., 2018) mutations have also been associated with drug resistance. More worryingly, plasmids harboring *tet(X3/X4/X5)* can confer high levels of tigecycline resistance in bacteria, posing a serious threat to the clinical effectiveness of this drug as a last line of defense (He et al., 2019; Sun et al., 2019; Wang et al., 2019). However, more and more tigecycline-resistant *Klebsiella pneumoniae* are being identified in the clinic.

Eravacycline, previously known as TP-434, is a novel fluorocycline antibiotic with broad-spectrum activity against Gram-positive and Gram-negative aerobic and anaerobic pathogens *in vitro*. It has been reported to be 2–4 times more effective than tigecycline against common clinical Gram-positive and Gram-negative aerobic bacteria (Zhanel et al., 2016).

The emergence of *tmexCD-toprJ* heralds the breach of the several classes of antibiotics, including the last group of antibiotics, tigecycline, by both chromosome-mediated and plasmid-mediated resistance. *TmexCD-toprJ* operon was the first plasmid-borne RND-type efflux pump that confers resistance to last-line antibiotics tigecycline and eravacycline. Notably, this operon can co-transfer with other mobile resistance determinants, including mcr-8, among *Enterobacteriaceae (* Lv et al., 2020; Sun et al., 2020). The genetic environment of *tmexCD-toprJ* is highly diverse and is associated with various mobile elements. Horizontal and vertical gene transfer of *tmexCD-toprJ* could have occurred, which might have led to its clinical dissemination (Dong et al., 2022).

In this study, we describe two CRKP strains, ZY308 and ZY309, characterized by carrying a novel plasmid-encoded resistance-nodulation-division (RND) efflux pump, *tmexCD-toprJ*, which is conferring resistance to multiple drugs, including tigecycline.

Acquired resistance genes are often transferred horizontally between bacteria by splicing transfer and other means with the help of mobile genetic elements such as plasmids and ICE (Li et al., 2018). The *tmexCD-toprJ* operon was present in both CRKP strains in the IncFIB(pNDM-Mar)/IncFII(Yp) type plasmid. Through the analysis, we found that four transfer function modules, oriT, Relaxase, T4CP and T4SS, existed on the IncFIB(pNDM-Mar)/IncFII(Yp) type plasmid. Meanwhile, by plasmid splicing assay, we successfully spliced the plasmid carrying the *tmexCD-toprJ* operon into Ec600, and the spliced bacteria showed tigecycline resistance. This confirms that the *tmexCD-toprJ* operon can be transmitted in the strain, which may be why CRKP carrying this operon were isolated from different patients during the same period of time. The above results suggest that this transmissibility may cause outbreaks of nosocomial infections within a short period of time. Previous studies from others have demonstrated that the major carbapenem resistance genes *blaKPC*-harboring plasmid type is IncFII plasmid (Mohamed et al., 2019). Interestingly, the co-existence of *tmexCD-toprJ* with *bla*_KPC_ in the same plasmid deserves much attention, as our conjugation assay and phylogenic analysis showed that the *tmexCD-toprJ-bla_KPC_* co-harboring plasmid is highly transferable and is a “super-resistant” plasmid (Sun et al., 2020). However, our study found that some bacteria had the *tmexCD-toprJ* operon but were sensitive to tigecycline/eravacycline, suggesting that not all bacteria acquiring this operon develop a resistant phenotype, possibly related to the expression of this operon by an unknown mechanism.

In addition, ZY308 and ZY309 showed a highly viscous phenotype, but by virulence experiments we found that the strains were not very virulent. A study from the United States has reported that clinical isolates with features of both multidrug-resistance and hypermucoviscosity have unexpectedly low virulence. This is consistent with our findings, in which the authors analyzed that the cause of this condition may be a mutation in the causative agent *rmpA*, but the exact mechanism is not known (Kochan et al., 2023).

Our current study still has many limitations. First, the small number of clinical bacteria carrying the *tmexCD-toprJ* operon in our collection makes it difficult to represent the clonal spread of *tmexCD-toprJ*-positive strains in local hospitals. Second, this was a retrospective study that lacked some results of prospective analysis. Finally, we did not elucidate the molecular mechanism by which the high expression of *tmexCD-toprJ* leads to multi-drug resistance, which needs to be further investigated.

Our results indicate that the *tmexCD-toprJ* operon can be transmitted on various plasmids in clinical *K. pneumoniae* strains. The spread of this operon in carbapenem-resistant *K. pneumoniae* strains may pose a significant threat to clinical infection control, as therapeutic options for this multidrug-resistant pathogen are totally limited.

## Data Availability

The datasets presented in this study can be found in online repositories. The names of the repository/repositories and accession number(s) can be found in the article/supplementary material.
